# Altered Biomarkers in Trophoblast Cells Obtained Noninvasively Prior to Clinical Manifestation of Perinatal Disease

**DOI:** 10.1038/srep32382

**Published:** 2016-09-23

**Authors:** Jay M. Bolnick, Hamid-Reza Kohan-Ghadr, Rani Fritz, Alan D. Bolnick, Brian A. Kilburn, Michael P. Diamond, D. Randall Armant, Sascha Drewlo

**Affiliations:** 1 Department of Obstetrics and Gynecology, Wayne State University School of Medicine, Detroit, MI, USA; 2 Department of Obstetrics and Gynecology, Georgia Regents University, Augusta, GA, USA; 3 Program in Reproductive and Adult Endocrinology, NIH, NICHD, DHHS, Bethesda, MD, USA.

## Abstract

A contributing factor to poor placental perfusion, leading to intrauterine growth restriction and preeclampsia, is the failure of invading extravillous trophoblast (EVT) cells to remodel the maternal uterine arteries during the first and second trimesters of pregnancy. Noninvasive assessment of EVT cells in ongoing pregnancies is possible beginning three weeks after conception, using trophoblast retrieval and isolation from the cervix (TRIC). Seven proteins were semi-quantified by immunofluorescence microscopy in EVT cells obtained between gestational weeks 6 and 20 from pregnancies with normal outcomes (N = 29) and those with intrauterine growth restriction or preeclampsia (N = 12). Significant differences were measured in expression of PAPPA, FLT1, ENG, AFP, PGF, and LGALS14, but not LGALS13 or the lineage marker KRT7. These findings provide for the first time direct evidence of pathology-associated protein dysregulation in EVT cells during early placentation. The TRIC platform provides a novel approach to acquire molecular signatures of EVT cells that can be correlated with pregnancy outcome.

Serum biomarker screening and uterine artery Doppler ultrasound are currently the standard of care for predicting intrauterine growth restriction (IUGR) and preeclampsia (PE) before the onset of maternal disease^1^. Biomarker proteins in the maternal blood, particularly those associated with angiogenesis and the stress response, become altered several weeks before clinical symptoms of PE or IUGR appear^2^. However, first trimester screening of multiple predictors remains uninformative before 11 weeks of gestation, even when combined in a multivariable model^3^. Thus, no reliable biomarkers are available to alert clinicians in the first trimester to pregnancies that will eventually develop IUGR or PE, impeding progress toward targeted management of high-risk pregnancies to lessen the impact on women and their fetuses.

Histological examination of placentas delivered by women with severe PE and IUGR suggests a prior disruption of EVT function in the first trimester that predisposes to uteroplacental insufficiency, characterized by reduced EVT invasion, inadequate remodeling of the spiral arteries, deferred elimination of endovascular trophoblastic plugs, and elevated EVT cell death^4^,^5^,^6^,^7^,^8^,^9^. IUGR and PE are syndromes that span a continuum of outcomes, ranging from mild to severe. Although it is thought that remodeling of the uterine arteries becomes deficient before PE and IUGR are diagnosed, direct evidence of early EVT dysfunction before the onset of clinical symptoms is lacking and the underlying cellular and molecular mechanisms remain unclear. There is evidence that circulating proteins altered in association with PE originate in the placental trophoblast cells, suggesting indirectly their involvement in the disease process^1^. Conditions arising later in gestation exacerbate the onset of disease, which can include endothelial cell dysfunction and a systemic maternal inflammatory response that can lead to organ failure^10^,^11^.

Although access to fetal cells for prenatal diagnosis is technically complex, and in some cases, such as amniocentesis or chorionic villous sampling, poses high risk^12^, a relatively simple solution to this challenge has been advanced over the past forty years^13^. The first report identifying fetal cells in the cervix of pregnant patients was described in 1971^14^. Following this landmark finding, multiple investigators have isolated trophoblast cells from the cervix of pregnant patients with varying degrees of success, ranging between 23–97%, depending on collections method, status of pregnancy, and gestational age^13^. Placental cells are shed into the cervical canal, but their clinical utility has been limited due to an excess of co-mingling maternal cells. Successful methods to obtain trophoblast cells during ongoing pregnancies include the antenatal cell extractor, cervical aspiration, endocervical canal lavage, intrauterine lavage, and transcervical smears with a cytobrush^13^. Although these methods are less invasive than conventional methods of obtaining intact fetal cells from ongoing pregnancies, safety of the above methods vary. Intrauterine lavage has been associated with limb reduction defects^15^. Obtaining trophoblast cells from the cervix of ongoing pregnancies with the same cytobrush used for a Papanicolaou test was attempted by multiple investigators^16^,^17^ and has been proven safe during pregnancy^18^,^19^,^20^,^21^.

The unique expression of human leukocyte antigen (HLA)-G on the surface of EVT cells, exclusive of adult tissues^22^, including maternal cervical cells^23^, can be used to distinguish fetal from maternal cells. We recently introduced trophoblast retrieval and isolation from the cervix (**TRIC**) to efficiently separate EVT cells in endocervical specimens from the resident maternal cells, using a monoclonal antibody to HLA-G^24^. Molecular characterization showed that the isolated cells have an EVT phenotype, based on expression of three trophoblast-specific proteins, β-subunit of human chorionic gonadotropin (hCGB), placental lactogen (CSH1), and cytokeratin 7 (KRT7); as well as five EVT-specific proteins, human leukocyte antigen G (HLA-G), VE-cadherin (CDH5), platelet endothelial cell adhesion molecule 1 (PECAM1), integrin-α1 (ITGA1) and matrix metalloproteinase 9 (MMP9); and the absence of three proteins exclusive to villous trophoblast, pregnancy specific beta-1-glycoprotein 1 (PSG1), integrin-α6 (ITGA6) and cadherin 1 (CDH1)^24^. TRIC provides approximately 1000 nearly homogeneous EVT cells for prenatal testing, beginning at 5 weeks gestational age, before it is currently possible to determine whether disease is developing. If EVT cells displaced to the endocervical canal are physiologically similar to those invading the uterine endometrium and vasculature, TRIC could provide a noninvasive approach to monitor the pathophysiology of pregnancy. When protein expression patterns were compared in EVT cells obtained by TRIC from pregnancies that ended in a miscarriage with gestational age-matched control pregnancies, significant differences were found^25^, suggesting that indeed EVT cells in the cervix reflect the physiological status of the pregnancy. Evidence indicates that developmental defects in EVT cells contribute to a large portion of miscarriages, as well as other disorders associated with uteroplacental insufficiency^9^. In cases where EVT dysfunction is common to miscarriage, IUGR and PE, similar protein changes would be expected in the first trimester.

We hypothesize that the molecular signatures of EVT cells early in gestation reflect their disrupted function in at least a portion of pregnancies that later develop IUGR or PE. Galectin 13 (LGALS13), galectin 14 (LGALS14), pregnancy-associated plasma protein-A (PAPPA), and placental growth factor (PGF) are proteins secreted by the placenta into the maternal circulation that decrease with abnormal placentation, prior to the onset of IURG or PE^26^,^27^,^28^,^29^,^30^. Elevated levels of soluble endoglin (sENG), soluble fms-like tyrosine kinase-1 (sFLT1), and alpha-fetoprotein (AFP), have been found in placentas or serum of patients with abnormal placentation^31^,^32^,^33^,^34^,^35^,^36^. Diseases linked to uteroplacental insufficiency share a similar trophoblast pathophysiology that generates abnormal placentation^9^. Therefore, expression of these seven proteins was evaluated in EVT cells obtained by TRIC prior to 20 weeks of gestational age to determine whether their levels are altered in women who later develop IUGR or PE.

## Material and Methods

### Patient selection

Endocervical specimens (N = 200) were collected with informed consent from women receiving initial prenatal care after spontaneous conception or infertility treatment at Wayne State University and Detroit Medical Center facilities. This study was carried out in accordance with the Declaration of Helsinki and all consent forms and protocols were approved by the Institutional Review Board of Wayne State University. Inclusion criteria specified women between 18 and 45 years of age, 5 to 20 weeks of gestation, both primagravida and multiparous females. Patients with multiple gestations or experiencing active vaginal bleeding were excluded from the study. Medical records were reviewed and women diagnosed with PE and/or IUGR were identified and selected (N = 12) for further study. IUGR was defined as estimated fetal weight less than the 10th percentile for gestational age^7^. PE was defined as new onset hypertension (blood pressure >140 systolic or >90 diastolic) and proteinuria (>1+ on a dipstick or >300 mg/24 h) at or after 20 weeks of gestation^37^. The control group selected from women sampled included gestational age-matched patients with an uncomplicated outcome and normal term delivery (N = 29). Patient demographics are summarized in Table 1.

### Endocervical Sampling

Endocervical specimens were obtained, as previously described^23^, using a ThinPrep kit (Hologic, Inc., Marlborough, MA) with cytobrush after placing a speculum in the vagina. Cervical mucus was included with the specimens. The cytobrush was rinsed into 20 ml of PreservCyt (Hologic) fixative solution and transported to the laboratory. Mucus was dissolved by adding 500 μl concentrated glacial acetic acid for 5 min to each specimen. Specimens were centrifuged at 400 × g for 5 min at 4 °C, the supernatant was removed, and the cell pellet was resuspended in 20 ml of ice-cold sterile phosphate buffered saline (PBS). The washing procedure was repeated twice and the final pellet was resuspended in 10 ml of PBS. To determine the content of trophoblast cells, a small aliquot of each processed specimen was centrifuged onto a slide using a Shandon Cytospin 3 centrifuge (Thermo-Fisher, Waltham, MA) at 1500 RPM for 5 min and labeled with 10 μg/ml of mouse anti-HLA-G (Clone 4H84, BD Biosciences, San Diego, CA or Clone G233, Exbio, Prague, Czech Republic), as previously described^23^.

### Isolation of Trophoblast Cells

The TRIC procedure was completed by combining cervical cells with mouse anti-HLA-G antibody bound to goat anti-mouse IgG conjugated to 250 nm magnetic nanoparticles (Clemente Associates, Madison, CT), and incubating overnight at 4 °C in 100 μl sterile PBS with mixing, as previously described^24^. Using a DynaMag™-Spin magnet (Life Technologies), the bound and non-bound cells were collected after three washes in 1 ml of PBS. About 1000 HLA-G-bound cells were recovered from each endocervical specimen (Table 1).

### Quality Controls for Purified EVT Cells

TRIC-isolated EVT cells were collected over a 12-month period and archived on slides. For ICC, small groups of approximately 50 cells were mounted on microscope slides by cytocentrifugation, inserting the end of a 100 μl (yellow) pipet tip into a Shandon Cytospin funnel to focus the cells within a 50-μm diameter area to facilitate imaging. The isolated cells were 97% positive for hCGB (Table 1), whereas the depleted cell fraction did not express this trophoblast marker^24^. It was previously reported that two other trophoblast marker proteins, cytokeratin 7 (KRT7) and placental lactogen (CSH1) are uniformly expressed in the isolated EVT cell fraction^24^. Fluorescence *in situ* hybridization with probes for the X and Y chromosomes reveals consistent XY or XX labeling of nuclei in agreement with the sex of babies delivered at the conclusion of pregnancy, verifying the fetal origin of cells isolated by TRIC^24^.

### Immunofluorescence Labeling

Microscope slides containing either isolated HLA-G-positive cells or recovered HLA-G-depleted cells were examined, using a standardized and quality controlled immunofluorescence protocol, with antibodies raised against hCGB, KRT7 (DAKO; Carpinteria, CA), LGALS13, LGALS14, PGF, PAPPA, AFP, ENG, or FLT-1 (R&D Systems). Antibodies were each titered to ensure a linear fluorescence signal with labeling, as previously reported^25^, and similar lots of antibody were used throughout the study. Primary antibody was diluted to 5 μg/ml in Tris-buffered saline containing 0.05% Tween-20 and 5 mg/ml BSA (TTBS/BSA), and incubated on slides for 17 hrs at 4 °C. Control cells from each specimen were incubated with 5 μg/ml of non-immune rabbit, goat or mouse IgG (Jackson Immuno Research, West Grove, PA), as appropriate. Slides were washed three times with TTBS/BSA and incubated for 1 h at room temperature in the dark with similar lots of FITC- or Texas Red-conjugated donkey anti-mouse, anti-rabbit, or anti-goat IgG (Jackson ImmunoResearch), as appropriate, diluted 1:250 in TTBS/BSA. After slides were washed three times with TTBS/BSA, nuclei were counterstained with 1 ng/ml of 4′,6-diamidino-2-phenylindole dihydrochloride (DAPI) for 10 min, followed by another three washes with TTBS/BSA. Slides were cover slipped with Vectashield mounting media (Vector Laboratories, Burlingame, CA) and sealed with nail polish. hCGB-positive and negative cells were counted by epifluorescence microscopy using a Leica DM IRB microscope (Leica Microsystems).

### Protein Quantification by Image Analysis

Each protein was quantified in individual EVT cells by immunofluorescence microscopy, using our published method^25^. Antibodies and nuclei were imaged at an objective magnification of 20 × with an exposure time of 2.0 seconds, using filter sets for DAPI, FITC and Texas Red. Images were captured with a Hamamatsu Orca cooled-chip digital camera. The FITC or Texas Red stain intensities were quantified using Simple PCI (Hamamatsu) imaging software. Fluorescence intensities (grey levels) were determined for each antibody and non-immune IgG (background) by choosing at least 3 fields per sample and circumscribing a minimum of 10 cells per field, resulting in a total of at least 30 cells assessed per sample.

The background values were averaged and subtracted from each fluorescent value. The background-subtracted values for each specimen were averaged to calculate the average fluorescent units (AFU). Each AFU value was divided by the average of the AFUs for the control cohort to generate the relative fluorescent unit (RFU) values. Slides were assessed together in a random fashion. KRT7, a trophoblast marker, was assessed in 17 of the specimens (9 controls and 8 adverse cases) to provide a technical and biological control for non-specific variances within the sample populations, assuming it was uniformly expressed in all EVT cells^24^.

### Statistical Analysis and Data Modeling

Statistical analyses were performed using JMP (version 10.0; SAS Institute, Cary, NC). Data were compared using the nonparametric Mann–Whitney *U*-test to evaluate statistical differences between groups, where *p* < 0.05 was considered significant.

Cluster analysis was performed and analyzed by BioNumerics (Applied Maths, Austin, TX, USA), using a hierarchical clustering algorithm with the Unweighted Pair Group Method with Arithmetic Mean (UPGMA). Recently, this technique has been used to produce clinically meaningful compact clusters^38^. Number of clusters was determined by dendrograms.

To calculate the accuracy of candidate protein markers in predicting adverse pregnancy outcomes, receiver operating characteristic (ROC) curves were employed and the areas under curves (AUCs) with 95% confidence intervals were calculated for each protein or combination of proteins.

## Results

Table 1 presents the demographic and obstetric characteristics of the study subjects. Maternal age was similar (approximately 28 years old) in both case and control groups. The pathological samples represented a range of preeclamptic phenotypes (mild to severe PE), as well as IUGR (below 10^th^ percentile). Specimens were obtained between 6 and 20 weeks of gestation at similar gestational ages (median = 13 and 13.5 for control and adverse cases, respectively). The purity of isolated cells (hCGB-positive) was similar in both groups (medians = 97%). The ratio of recovered fetal cells to maternal cells was similar in adverse gestations (median = 65 × 10^−5^) compared to controls (median = 54 × 10^−5^).

**Figure 1:**
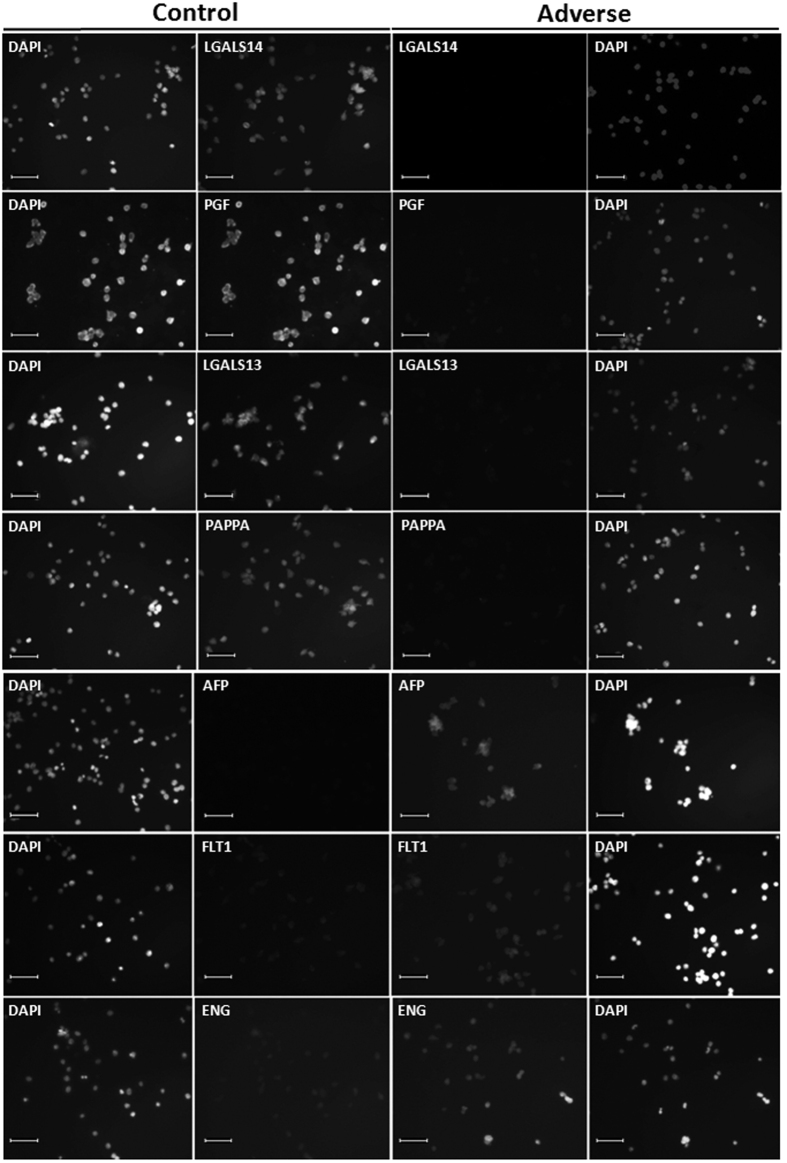
Representative fluorescence microscopic images of EVT cells obtained by TRIC from Control and Adverse pregnancies. Paired images are shown for fields of cells labeled with DAPI or by immunofluorescence for the proteins, as indicated. Size bars, 100 μm.

**Figure 2:**
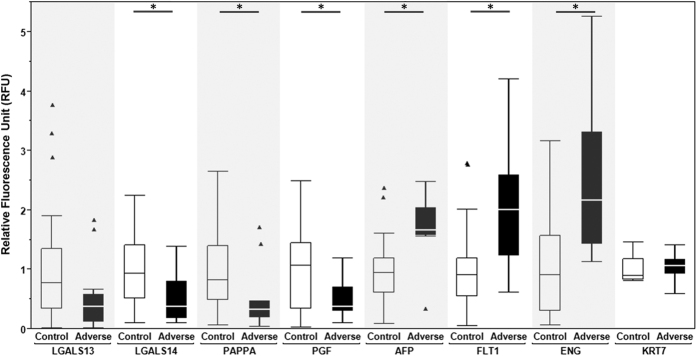
Box-and-whisker plot showing the distribution of normalized Relative Fluorescence Unit values of each candidate protein biomarker for control (white boxes) and adverse outcome (black boxes) patients. The boxes represent the 25^th^ to 75^th^ percentiles, and horizontal lines within the boxes indicate the medians. The whiskers are drawn to indicate 1.5 × Inter Quartile Range (3rd quartile – 1st quartile). The triangles represent outliers. *p < 0.05 between control and adverse cohorts for each protein. For KRT7, N = 9 for control, N = 8 for adverse. For all other proteins, N = 29 for control, N = 12 for adverse.

Seven candidate biomarkers for adverse pregnancy outcome and the trophoblast-specific intermediate filament protein KRT7 were analyzed by standardized immunofluorescence microscopy to derive RFU values, as detailed in the Materials and Methods section (Figs 1 and 2). LGALS13 expression was reduced in most adverse cases (e.g., Fig. 1); however, due to high variability, the difference was not statistically significant (Fig. 2). LGALS14 was notably reduced (median (IQR) = 0.48 (0.22–0.84)) in EVT cells from pathological pregnancies compared to healthy controls (median (IQR) = 0.91 (0.50–1.37)). Both PGF and PAPPA were highly expressed (median (IQR) = 1.07 (0.30–1.46) and 0.81(0.48–1.41) respectively) in EVT cells recovered from healthy pregnancies compared to complicated counterparts (median (IQR) = 0.38 (0.30–0.71) and 0.4 (0.22–0.46), respectively). AFP, FLT1, and ENG were all highly expressed in adverse pregnancies (median (IQR): 1.76 (1.60–2.06), 2.09 (1.49–2.63), and 2.17 (1.43–3.32), respectively), as compared to the control group (median (IQR): 0.94 (0.62–1.26), 0.92 (0.55–1.19, and 0.92 (0.33–1.59), respectively). Levels of KRT7 were similar (p = 0.85) in samples examined from both groups (Table 2 and Fig. 2), supporting the specificity of these findings. The sample size precluded determination of whether gestational age influenced the correlation between protein RFU levels and pregnancy outcome. However, the distribution of the data gave no indication that there was a trend dependent on gestation (Supplementary Fig. S1).

**Figure 3:**
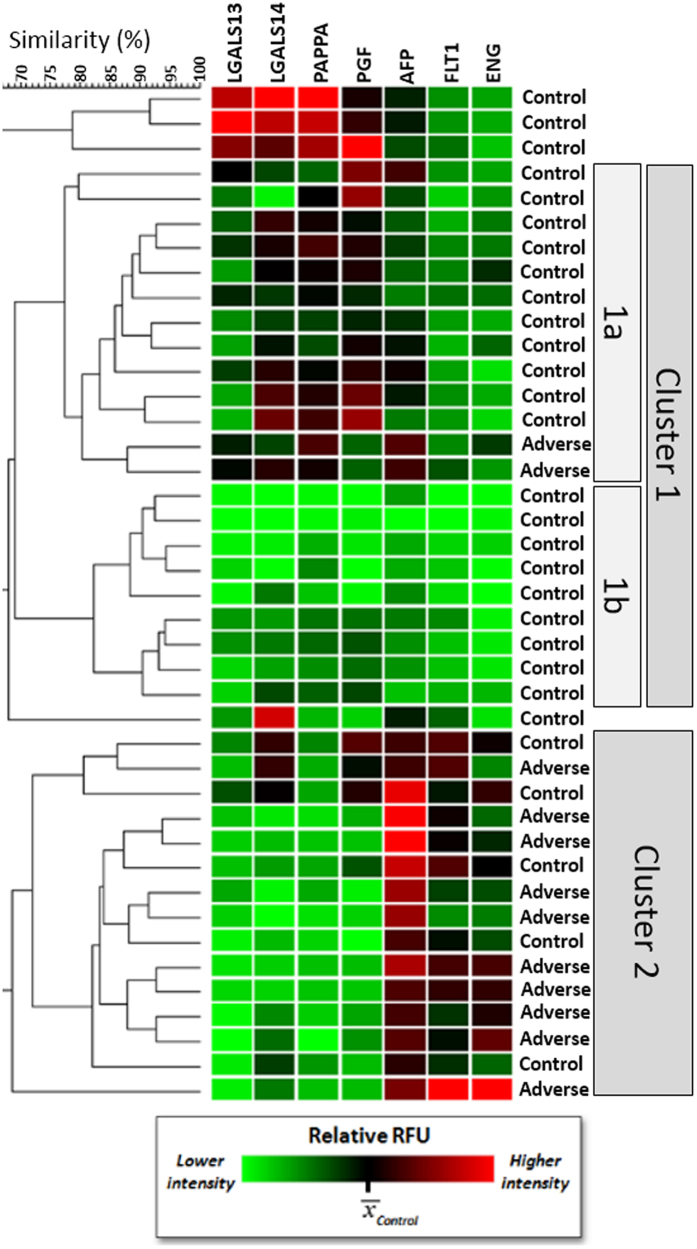
Hierarchical clustering analysis. Two major clusters were identified and are indicated as Cluster1 and Cluster2. Cluster1 was further subdivided into two sub-clusters (**1a,1b**) with distinguishable expression patterns. A third subgroup of three control pregnancies showing an “extreme” normal profile were clustered separately at the top of the chart, and have a high degree of similarity (78.8%).

We used a hierarchical clustering algorithm to determine whether the two pregnancy outcome groups (healthy versus adverse) were distinguishable. In this model (Fig. 3), Cluster 1 mostly includes healthy control samples (92%) with a minimum similarity of 68%, and contains two major sub-clusters with two distinct biomarker expression patterns. One is characterized by over-expression of LGALS13, LGALS14, PAPPA and PGF (1a), and the other is described by under-expression of all seven protein (1b). Cluster 2 contains 67% adverse pregnancy outcomes with a minimum similarity of 68.7% (Fig. 3). Despite the diversity of clinical phenotypes generated by uteroplacental insufficiency, the selected biomarkers successfully identified two main clusters distinguishing healthy and adverse pregnancy outcomes. To test whether a combination of factors correlates better with pregnancy outcome, a series of ROC curves were generated for all combinations of biomarkers, and the areas under the curves (AUCs) were calculated and plotted (Supplementary Fig. S2). The highest AUC (0.948) was achieved with all markers present in the model (AUC = 0.95; Supplementary Fig. S2-B, red bar). Equivalent AUCs were achieved with certain combinations of 4–6 marker, which each included PGF (AUC = 0.95; Supplementary Fig. S2-B, pink shaded zone). The lowest AUCs were obtained with combinations that lacked the three proteins that increased with adverse outcomes (ENG, FLT1 and AFP).

## Discussion

Real-time, noninvasive assessment of EVT cells in ongoing pregnancies is now possible beginning at five weeks of gestation (three weeks after conception), using TRIC^24^. The present study, for the first time, assessed and documented the association between the protein expression profile of cervical EVT cells recovered by TRIC and later development of IUGR or PE. Cells isolated by TRIC express proteins consistent with the extravillous trophoblast lineage, and that differ markedly from villous trophoblast^24^. Although EVT cells obtained by TRIC reside in a locale other than the placenta, the ability to correlate changes in protein biomarker profiles with pregnancy disorders suggests that they are relevant to placental health. The mechanism of EVT migration into the endocervical canal remains enigmatic.

The assessment of EVT cells obtained by TRIC for known circulating biomarkers with potential correlation to pregnancy complications could fill the existing gap in knowledge about the contribution of dysregulated early EVT development to the underlying etiologic cellular and molecular mechanisms of IUGR and PE. Galectin-13 (LGALS13/PP13) and LGALS14 are among the placenta-specific galectins of mammals^39^. The observed lower expression of both galectins in pathological EVT cells accords with their significant decrease in maternal serum during the first trimester in women who later develop early-onset PE^27^,^40^. Serum markers used to screen for aneuploidy (AFP and PAPPA) were also altered in EVT fetal cells from pathologic pregnancies, supporting the reliability of these factors for early prediction of pregnancies at risk for IUGR and PE shown in previous reports^41^,^42^,^43^. In a recent report^25^, we found comparable differences in protein marker expression patterns in EVT cells obtained by TRIC from women with an early pregnancy loss, supporting the hypothesis^9^ of EVT cell disruption preceding diseases of severe placental insufficiency that can, depending on other unknown factors, catastrophically terminate pregnancy, reduce fetal growth or develop into PE. Our findings suggest that an intrinsic molecular defect in EVT cells occurs early in gestation that disrupts placental development, and is retained by EVT cells after displacement from the uteroplacental environment. Whether the molecular signatures of EVT cells in the endocervical canal accurately represent those within the placenta awaits experimental evidence that directly compares the two populations.

There is encouraging evidence for the utility of circulating sFLT1, sENG and PGF as biomarkers several weeks before clinical signs of PE appear^44^,^45^,^46^,^47^. In the second stage of disease progression, anti-angiogenic factors accumulate in maternal blood, particularly soluble splice variants of FLT1 and ENG^2^. Pro-angiogenic proteins, like PGF, are reduced, and receptor binding is antagonized by sFLT1. Both serum biomarker screening and uterine artery Doppler ultrasound have been explored for prediction of uteroplacental insufficiency before maternal disease appears^48^,^49^. Screening the protein levels of these markers in EVT cells from first and second trimester pregnancies revealed significant alterations, either increasing (FLT1 and ENG) or decreasing (PGF) in pregnancies that later developed PE or IUGR (Figs 1 and 2), similar to what has been described in maternal serum^44^. Overall, we found that the expression patterns of candidate biomarkers for adverse pregnancy outcomes in EVT cells recapitulated what has been described in serum at later stages of disease, suggesting that the EVT phenotype is established very early in pregnancy.

Clustering analysis demonstrated that, within the population of patients studied, 79% of the healthy pregnancies (N = 23) partitioned into a distinct group composed of 92% with uncomplicated outcomes (Fig. 3). These findings confirm that the altered maternal serum protein levels observed in women who develop PE or IUGR reflect changes in the EVT cells. PE and IUGR are syndromes with complex etiologies that are not strictly the result of uteroplacental insufficiency^50^. Therefore, it is not surprising that two adverse pregnancies partitioned with the cluster of mostly normal pregnancies and five normal cases were distributed throughout the cluster that contained 67% adverse outcomes. These apparent misplacements could result from limitations of the biomarker analyses, or reflect uteroplacental insufficiency that did not culminate in clinical disease. Doppler ultrasonographic data was not available to investigate uteroplacental perfusion. Numerous proteins fluctuate in their abundance in association with PE and IUGR, including proteins involved in cell adhesion, vascular development and oxidative stress^9^,^51^, that could be surveyed in cells obtained by TRIC, which might be superior in identifying pregnancies at risk than those selected in this study. We selected seven proteins of placental origin that accumulate in maternal serum and have been shown to fluctuate according to pregnancy outcome, finding that expression of six of the seven proteins were significantly altered in EVT cells collected at 6–20 weeks of gestation in pregnancies that developed perinatal disease after 20 weeks. ROC analysis demonstrated that sensitivity and specificity were enhanced by considering multiple biomarkers, rather than single protein levels. The area under the curve reached nearly 95% when 4 to 7 proteins were used simultaneously. This suggests a considerably stronger correlation with pregnancy outcome than has been reported using serum levels of these or other proteins that can be accessed noninvasively.

First trimester screening of multiple predictors is currently uninformative even when combined in a multivariable model^3^. The inability to recognize pathologies of uteroplacental insufficiency early in pregnancy, before overt clinical symptoms arise, has been linked to limitations in current noninvasive interrogation of the placenta^52^. EVT cells obtained through TRIC provide a noninvasive tool to investigate the expression of candidate protein biomarkers and their relationship to pregnancy outcome. Beyond the direct health benefits for mothers and babies at risk, the development of valid biomarkers to identify healthy pregnancies could foster personalized prenatal care and channel resources towards the treatment of high-risk pregnancies.

## Conclusion

Safe access to intact fetal cells throughout pregnancy is considered the “Holy Grail” of perinatal diagnosis. The molecular signature of EVT cells from the placenta can now be monitored noninvasively in real-time, using TRIC and downstream analyses as demonstrated here with semi-quantitative immunohistochemistry. Candidate biomarkers quantified in EVT cells captured through TRIC have successfully distinguished healthy from adverse pregnancies, introducing a new era of perinatal screening to manage fetal and maternal health.

## Additional Information

**How to cite this article**: Bolnick, J. M. *et al*. Altered Biomarkers in Trophoblast Cells Obtained Noninvasively Prior to Clinical Manifestation of Perinatal Disease. *Sci. Rep.* **6**, 32382; doi: 10.1038/srep32382 (2016).

## Supplementary Material

Supplementary Information

## Figures and Tables

**Table 1:** Demographic information of studied subjects. (IQR = interquartile range).

	Control	Adverse
Number, *n (% of total)*	29 (70)	12 (30)
Maternal age in years, *median (IQR)*	28 (24.5–30)	28.5 (24–31)
Ethnicity		
Caucasian, *n (%)*	3 (10)	2 (17)
African, *n (%)*	18 (62)	7 (58)
Asian, *n (%)*	1 (3)	0 (0)
Hispanic, *n (%)*	0 (0)	1 (8)
Unknown, *n (%)*	7 (24)	2 (17)
Gestational age at the time of sampling in weeks, *median (IQR)*	13 (11–17)	13.5 (11.25–17)
Gestational age at delivery in weeks, *median (IQR)*	39 (38–40)	37 (34.25–38)
Gravity, *median (IQR)*	2 (1–3.25)	3 (1–5)
Parity		
Nulliparous, *n (%)*	11 (38)	4 (33)
Parous, *n (%)*	17 (58)	8 (67)
Unknown, *n (%)*	1 (4)	0 (0)
Delivery mode		
Normal Spontaneous Vaginal Delivery (NSVD). *n (%)*	22 (76)	6 (50)
Cesarean section (CS). *n (%)*	7 (24)	6 (50)
Birth Weight (gr), *median (IQR)*	3082 (2847–3521.25)	2402.5 (1900–2499)
Sex		
Male, *n (%)*	15 (52)	6 (50)
Female, *n (%)*	12 (41)	6 (50)
Unknown, *n (%)*	2 (7)	0 (0)
Adverse pregnancy outcomes, *n (%)*		
IUGR, *n (%)*		6 (50)
IUGR/sPE, *n (%)*		2 (17)
sPE, *n (%)*		4 (33)
Recovered fetal cells		
Count, *median (IQR)*	1030 (712.5–1113.7)	529 (460.5–928.8)
Fetal/Maternal ratio, *median (IQR)*	54E-5(29E-5–82E-5)	65E-5(47E-5–121E-5)
Purity (percentage of hCGB positive cells), *median (IQR)*	97.7 (94.9–98.9)	96.6 (94.3–100)

**Table 2:** The Relative Fluorescence Unit (RFU) values for studied biomarkers.

Protein					Normal approximation	
	Control		Adverse		(Two-tailed)	
	RFU Median	CV (%)	RFU Median	CV (%)	Z	Prob > |Z|
	(IQR)		(IQR)			
LGALS13	0.77 (0.33–1.37)	135.1	0.38 (0.16–0.62)	135.1	−1.61892	0.1055
LGALS14	0.91 (0.50–1.37)	95.6	0.48 (0.22–0.84)	129.2	−1.99141	0.0464*
PAPPA	0.81 (0.48–1.41)	114.8	0.34 (0.22–0.46)	70.6	−2.62179	0.0087*
PGF	1.07 (0.30–1.46)	108.4	0.38 (0.30–0.71)	107.9	−2.10603	0.0352*
AFP	0.94 (0.62–1.26)	68.1	1.76 (1.60–2.06)	26.1	4.25503	<0.0001*
FLT1	0.92 (0.55–1.19)	69.6	2.09 (1.49–2.63)	54.5	3.596	0.0003*
ENG	0.92 (0.33–1.59)	137.0	2.17 (1.43–3.32)	87.1	3.5102	0.0004*
KRT7	0.87 (0.83–0.99)	18.4	0.98 (0.91–1.16)	25.5	0.81791	0.4134

Coefficient of Variation (CV) was estimated as IQR/median and is presented as percentage. The Mann-Whitney U test was also performed to compare the difference between two groups. The Z value for two-tailed normal approximation was calculated from the U value, *p < 0.05.

## References

[b1] Grill S. . Potential markers of preeclampsia–a review. Reproductive biology and endocrinology: RB&E 7, 70, 10.1186/1477-7827-7-70 (2009). 19602262 PMC2717076

[b2] Maynard S., Epstein F. H. & Karumanchi S. A. Preeclampsia and angiogenic imbalance. Annu. Rev. Med. 59, 61–78, 10.1146/annurev.med.59.110106.214058 (2008). 17937587

[b3] Myatt L. . The utility of uterine artery Doppler velocimetry in prediction of preeclampsia in a low-risk population. Obstet. Gynecol. 120, 815–822, 10.1097/AOG.0b013e31826af7fb (2012). 22996099 PMC3449210

[b4] Khong T. Y., De Wolf F., Robertson W. B. & Brosens I. Inadequate maternal vascular response to placentation in pregnancies complicated by pre-eclampsia and by small-for- gestational age infants. Br. J. Obstet. Gynaecol. 93, 1049–1059 (1986). 3790464 10.1111/j.1471-0528.1986.tb07830.x

[b5] Brosens I. A., Robertson W. B. & Dixon H. G. The role of the spiral arteries in the pathogenesis of preeclampsia. Obstet. Gynecol. Annu. 1, 177–191 (1972). 4669123

[b6] DiFederico E., Genbacev O. & Fisher S. J. Preeclampsia is associated with widespread apoptosis of placental cytotrophoblasts within the uterine wall. Am. J. Pathol. 155, 293–301 (1999). 10393861 10.1016/S0002-9440(10)65123-1 PMC1866652

[b7] Ishihara N. . Increased apoptosis in the syncytiotrophoblast in human term placentas complicated by either preeclampsia or intrauterine growth retardation. Am. J. Obstet. Gynecol. 186, 158–166 (2002). 11810103 10.1067/mob.2002.119176

[b8] Allaire A. D., Ballenger K. A., Wells S. R., McMahon M. J. & Lessey B. A. Placental apoptosis in preeclampsia. Obstet. Gynecol. 96, 271–276 (2000). 10908776 10.1016/s0029-7844(00)00895-4

[b9] Burton G. J. & Jauniaux E. Placental oxidative stress: from miscarriage to preeclampsia. J. Soc. Gynecol. Investig. 11, 342–352, 10.1016/j.jsgi.2004.03.003S1071-5576(04)00138-8 (2004). 15350246

[b10] Roberts J. M. Endothelial dysfunction in preeclampsia. Semin. Reprod. Endocrinol. 16, 5–15 (1998). 9654603 10.1055/s-2007-1016248

[b11] Redman C. W. & Sargent I. L. Placental debris, oxidative stress and pre-eclampsia. Placenta 21, 597–602 (2000). 10985960 10.1053/plac.2000.0560

[b12] Wapner R. J. Invasive prenatal diagnostic techniques. Semin. Perinatol. 29, 401–404, 10.1053/j.semperi.2006.01.003 (2005). 16533654

[b13] Imudia A. N., Kumar S., Diamond M. P., Decherney A. H. & Armant D. R. Transcervical retrieval of fetal cells in the practice of modern medicine: a review of the current literature and future direction. Fertil. Steril. 93, 1725–1730, S0015-0282(09)04051-5 10.1016/j.fertnstert.2009.11.022 (2010). 20056202 PMC2847626

[b14] Shettles L. B. Use of the Y chromosome in prenatal sex determination. Nature 230, 52–53 (1971). 4102825 10.1038/230052b0

[b15] Chou M. M., Lin S. K. & Ho E. S. Severe limb reduction defects after uterine lavage at 7-8 weeks’ gestation. Prenatal diagnosis 17, 77–80 (1997). 9021832

[b16] Kingdom J., Sherlock J., Rodeck C. & Adinolfi M. Detection of trophoblast cells in transcervical samples collected by lavage or cytobrush. Obstetrics and gynecology 86, 283–288 (1995). 7617362 10.1016/0029-7844(95)00127-d

[b17] Fejgin M. D., Diukman R., Cotton Y., Weinstein G. & Amiel A. Fetal cells in the uterine cervix: a source for early non-invasive prenatal diagnosis. Prenatal diagnosis 21, 619–621 (2001). 11536257 10.1002/pd.117

[b18] Paraiso M. F., Brady K., Helmchen R. & Roat T. W. Evaluation of the endocervical Cytobrush and Cervex-Brush in pregnant women. Obstet. Gynecol. 84, 539–543 (1994). 8090390

[b19] Holt J., Stiltner L., Jamieson B. & Fashner J. Clinical inquiries. Should a nylon brush be used for Pap smears from pregnant women? J. Fam. Pract. 54, 463–464, jfp_0505_5405n (2005). 15865907

[b20] Orr J. W. Jr., Barrett J. M., Orr P. F., Holloway R. W. & Holimon J. L. The efficacy and safety of the cytobrush during pregnancy. Gynecol. Oncol. 44, 260–262 (1992). 1541438 10.1016/0090-8258(92)90053-l

[b21] Rivlin M. E. . Comparison of cytobrush and cotton swab for Papanicolaou smears in pregnancy. J. Reprod. Med. 38, 147–150 (1993). 8445608

[b22] Loke Y. W. . Evaluation of trophoblast HLA-G antigen with a specific monoclonal antibody. Tissue Antigens 50, 135–146 (1997). 9271823 10.1111/j.1399-0039.1997.tb02852.x

[b23] Imudia A. N. . Retrieval of trophoblast cells from the cervical canal for prediction of abnormal pregnancy: a pilot study. Hum. Reprod. 24, 2086–2092, dep206 10.1093/humrep/dep206 (2009). 19497946 PMC2727404

[b24] Bolnick J. M. . Trophoblast retrieval and isolation from the cervix (TRIC) for noninvasive prenatal screening at 5 to 20 weeks of gestation. Fertil. Steril. 102, 135-142 e136, 10.1016/j.fertnstert.2014.04.008 (2014). PMC10411519 24825422

[b25] Fritz R. . Noninvasive detection of trophoblast protein signatures linked to early pregnancy loss using trophoblast retrieval and isolation from the cervix (TRIC). Fertil. Steril. 104, 339-346 e334, 10.1016/j.fertnstert.2015.05.010 (2015). PMC4522365 26051097

[b26] Huppertz B., Meiri H., Gizurarson S., Osol G. & Sammar M. Placental protein 13 (PP13): a new biological target shifting individualized risk assessment to personalized drug design combating pre-eclampsia. Human reproduction update 19, 391–405, 10.1093/humupd/dmt003 (2013). 23420029

[b27] Than N. G. . Evolutionary origins of the placental expression of chromosome 19 cluster galectins and their complex dysregulation in preeclampsia. Placenta 35, 855–865, 10.1016/j.placenta.2014.07.015 (2014). 25266889 PMC4203431

[b28] Smith G. C. . Early pregnancy levels of pregnancy-associated plasma protein a and the risk of intrauterine growth restriction, premature birth, preeclampsia, and stillbirth. J. Clin. Endocrinol. Metab. 87, 1762–1767 (2002). 11932314 10.1210/jcem.87.4.8430

[b29] Nucci M., Poon L. C., Demirdjian G., Darbouret B. & Nicolaides K. H. Maternal Serum Placental Growth Factor (PlGF) Isoforms 1 and 2 at 11-13 Weeks’ Gestation in Normal and Pathological Pregnancies. Fetal diagnosis and therapy, 10.1159/000357842 (2014). 24457972

[b30] Ghosh S. K., Raheja S., Tuli A., Raghunandan C. & Agarwal S. Can maternal serum placental growth factor estimation in early second trimester predict the occurrence of early onset preeclampsia and/or early onset intrauterine growth restriction? A prospective cohort study. The journal of obstetrics and gynaecology research 39, 881–890, 10.1111/jog.12006 (2013). 23496304

[b31] Farina A. . Gene expression in chorionic villous samples at 11 weeks’ gestation from women destined to develop preeclampsia. Prenatal diagnosis 28, 956–961, 10.1002/pd.2109 (2008). 18792924

[b32] Levine R. J. . Circulating angiogenic factors and the risk of preeclampsia. N. Engl. J. Med. 350, 672–683, 10.1056/NEJMoa031884 (2004). 14764923

[b33] Chung J. Y., Song Y., Wang Y., Magness R. R. & Zheng J. Differential expression of vascular endothelial growth factor (VEGF), endocrine gland derived-VEGF, and VEGF receptors in human placentas from normal and preeclamptic pregnancies. The Journal of clinical endocrinology and metabolism 89, 2484–2490, 10.1210/jc.2003-031580 (2004). 15126581 PMC3282114

[b34] Williams M. A. . Elevated maternal serum alpha-fetoprotein levels and midtrimester placental abnormalities in relation to subsequent adverse pregnancy outcomes. American journal of obstetrics and gynecology 167, 1032–1037 (1992). 1384333 10.1016/s0002-9378(12)80033-0

[b35] Waller D. K., Lustig L. S., Cunningham G. C., Feuchtbaum L. B. & Hook E. B. The association between maternal serum alpha-fetoprotein and preterm birth, small for gestational age infants, preeclampsia, and placental complications. Obstetrics and gynecology 88, 816–822 (1996). 8885920 10.1016/0029-7844(96)00310-9

[b36] Lai J., Syngelaki A., Poon L. C., Nucci M. & Nicolaides K. H. Maternal serum soluble endoglin at 30-33 weeks in the prediction of preeclampsia. Fetal Diagn Ther 33, 149–155, 10.1159/000343220 (2013). 23154616

[b37] Milne F. . The pre-eclampsia community guideline (PRECOG): how to screen for and detect onset of pre-eclampsia in the community. BMJ 330, 576–580, 10.1136/bmj.330.7491.576 (2005). 15760998 PMC554032

[b38] Stanek J. & Biesiada J. Clustering of maternal-fetal clinical conditions and outcomes and placental lesions. American journal of obstetrics and gynecology 206, 493.e491–e498, 10.1016/j.ajog.2012.03.025 (2012). 22534079

[b39] Than N. G. . A primate subfamily of galectins expressed at the maternal-fetal interface that promote immune cell death. Proc. Natl. Acad. Sci. USA. 106, 9731–9736, 10.1073/pnas.0903568106 (2009). 19497882 PMC2689813

[b40] Romero R. . First-trimester maternal serum PP13 in the risk assessment for preeclampsia. American journal of obstetrics and gynecology 199, 122 e121–122 e111, 10.1016/j.ajog.2008.01.013 (2008). 18539259 PMC2784814

[b41] Morris R. K. . Serum screening with Down’s syndrome markers to predict pre-eclampsia and small for gestational age: systematic review and meta-analysis. BMC pregnancy and childbirth 8, 33, 10.1186/1471-2393-8-33 (2008). 18680570 PMC2533288

[b42] Conde-Agudelo A., Papageorghiou A. T., Kennedy S. H. & Villar J. Novel biomarkers for predicting intrauterine growth restriction: a systematic review and meta-analysis. BJOG 120, 681–694, 10.1111/1471-0528.12172 (2013). 23398929

[b43] Metcalfe A., Langlois S., Macfarlane J., Vallance H. & Joseph K. S. Prediction of obstetrical risk using maternal serum markers and clinical risk factors. Prenat. Diagn. 34, 172–179, 10.1002/pd.4281 (2014). 24226970

[b44] Hagmann H., Thadhani R., Benzing T., Karumanchi S. A. & Stepan H. The promise of angiogenic markers for the early diagnosis and prediction of preeclampsia. Clin. Chem. 58, 837–845, 10.1373/clinchem.2011.169094 (2012). 22431894

[b45] Gao J. . [Value of second trimester maternal serum sFlt-1, PlGF and their ratio in the prediction of preeclampsia]. Zhonghua Fu Chan Ke Za Zhi 49, 22–25 (2014). 24694913

[b46] Prefumo F. Re: Uterine artery Doppler and sFlt-1/PlGF ratio: prognostic value in early-onset pre-eclampsia. Gomez-Arriaga, P. I., Herraiz, I., Lopez-Jimenez, E. A., Escribano, D., Denk, B. and Galindo, A. Ultrasound Obstet Gynecol 2014; 43: 525-532. *Ultrasound Obstet Gynecol* **43**, 488-489, 10.1002/uog.13362 (2014). 24789304

[b47] Allen R. E., Rogozinska E., Cleverly K., Aquilina J. & Thangaratinam S. Abnormal blood biomarkers in early pregnancy are associated with preeclampsia: a meta-analysis. Eur J Obstet Gynecol Reprod Biol 182, 194–201, 10.1016/j.ejogrb.2014.09.027 (2014). 25305662

[b48] Hossain N. & Paidas M. J. Adverse pregnancy outcome, the uteroplacental interface, and preventive strategies. Semin. Perinatol. 31, 208–212, 10.1053/j.semperi.2007.05.002 (2007). 17825674

[b49] Papageorghiou A. T., Yu C. K. & Nicolaides K. H. The role of uterine artery Doppler in predicting adverse pregnancy outcome. Best Pract Res Clin Obstet Gynaecol 18, 383–396, 10.1016/j.bpobgyn.2004.02.003 (2004). 15183134

[b50] Redman C. W. & Sargent I. L. Latest advances in understanding preeclampsia. Science (Wash.DC) 308, 1592–1594 (2005). 10.1126/science.1111726 15947178

[b51] Zhou Y., Damsky C. H. & Fisher S. J. Preeclampsia is associated with failure of human cytotrophoblasts to mimic a vascular adhesion phenotype. One cause of defective endovascular invasion in this syndrome? J. Clin. Invest. 99, 2152–2164 (1997). 9151787 10.1172/JCI119388 PMC508045

[b52] Guttmacher A. E., Maddox Y. T. & Spong C. Y. The Human Placenta Project: placental structure, development, and function in real time. Placenta 35, 303–304, 10.1016/j.placenta.2014.02.012 (2014). 24661567 PMC3999347

