# Supplementary material for: Altered Biomarkers in Trophoblast Cells Obtained Noninvasively Prior to Clinical Manifestation of Perinatal Disease
Source: Sci Rep. 2016 Sep 23;6:32382. doi: 10.1038/srep32382 (PMC5034887; doi:10.1038/srep32382)
Supplement: Supplementary Information [file srep32382-s1.pdf]

Altered Biomarkers in Trophoblast Cells Obtained Noninvasively Prior to Clinical Manifestation of Perinatal Disease

Jay M. Bolnick*[a], Hamid-Reza Kohan-Ghadr*[a], Rani Fritz[a], Alan D. Bolnick[a], Brian A. Kilburn[a], Michael P. Diamond[b], D. Randall Armant[a,c], Sascha Drewlo[a]

[a] Department of Obstetrics and Gynecology, Wayne State University School of Medicine, Detroit, MI; [b] Department of Obstetrics and Gynecology, Georgia Regents University, Augusta, GA; [c] Program in Reproductive and Adult Endocrinology, NIH, NICHD, DHHS, Bethesda, MD

*Contributed equally to the study.

Correspondence should be addressed to: Sascha Drewlo, Ph.D.
Department of Obstetrics and Gynecology
C.S. Mott Center, Wayne State University
275 E. Hancock Street, Detroit, MI 48201
Tel: 313-577-1158; Fax: 313-577-8554
Email: sdrewlo@Med.Wayne.edu

Supplementary Figure S1. Protein profiles for fetal cells obtained by TRIC from control pregnancies plotted against the gestational age at the time cervical samples were collected. Relative Fluorescence Unit values determined for the indicated proteins were calculated as described in the Materials and Methods section. Where multiple samples were obtained in the same week, error bars indicate the SEM. No significant trends were observed, and the regression lines were drawn for each profile.

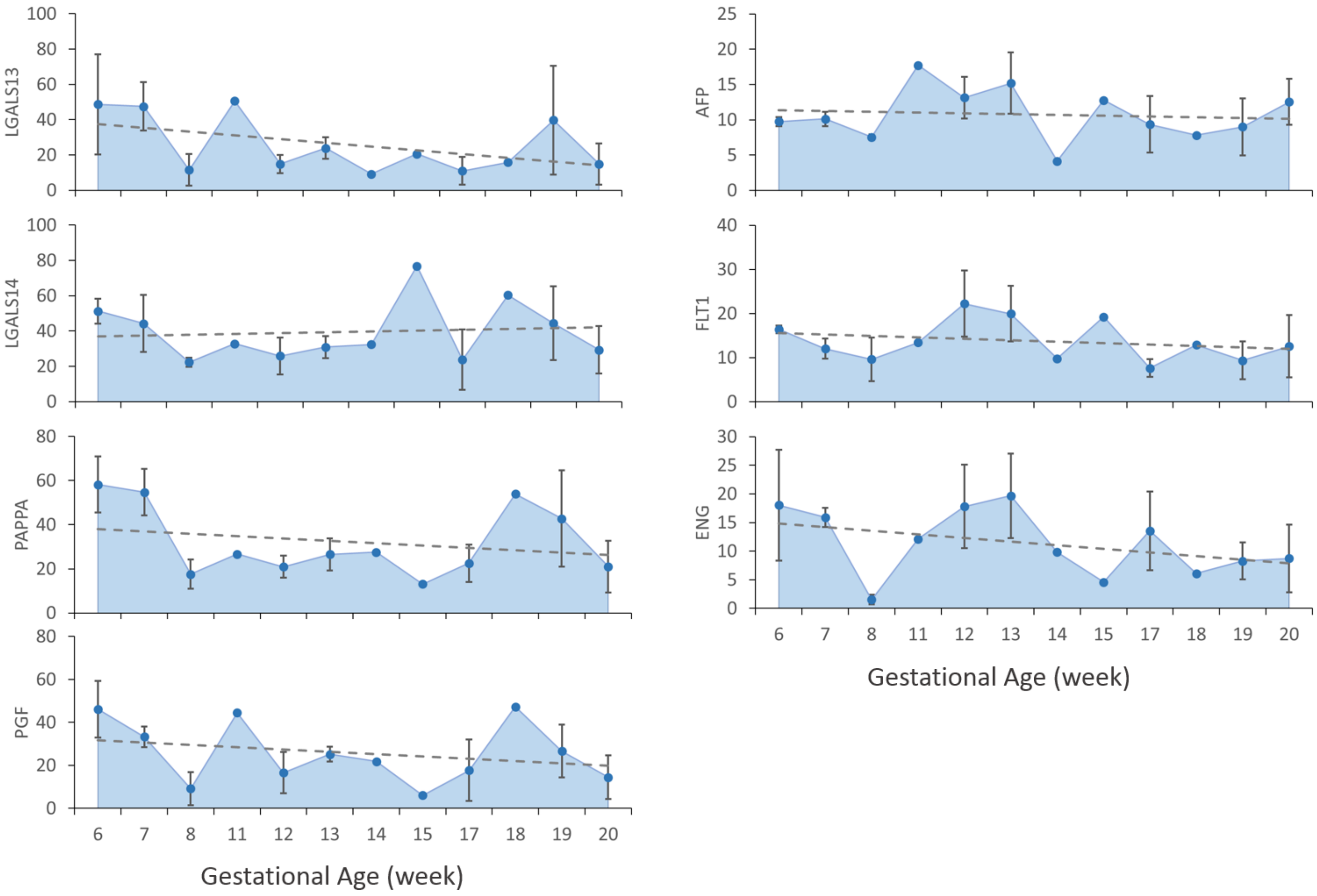

Supplementary Figure S2. Receiver operating characteristic curve analysis of biomarker combinations. All possible combinations of the seven biomarkers were analyzed by ROC to evaluate the accuracy of the tests. After determining the area under the curve (AUC) and corresponding 95% confidence intervals (CI), ROC curves for five of combinations are shown, ordered according to ascending AUCs (A). The sorted AUC values of all combinations are visualized as a histogram, where each bar represents the calculated AUC for one set from the combination matrix, represented below **(B)**. Along the x-axis, the inclusion of the protein markers in each combination set is illustrated by filling in the corresponding space. Each of the colored bars represent one of the ROC curves in upper panel (A) drawn in the same color. Among the last 8 combination sets with equally high AUCs (0.95; shaded in pink), a 4-factor (LGALS13, LGALS14, PGF, FLT1) combination had the fewest components with the highest sensitivity and specificity (magenta bar).

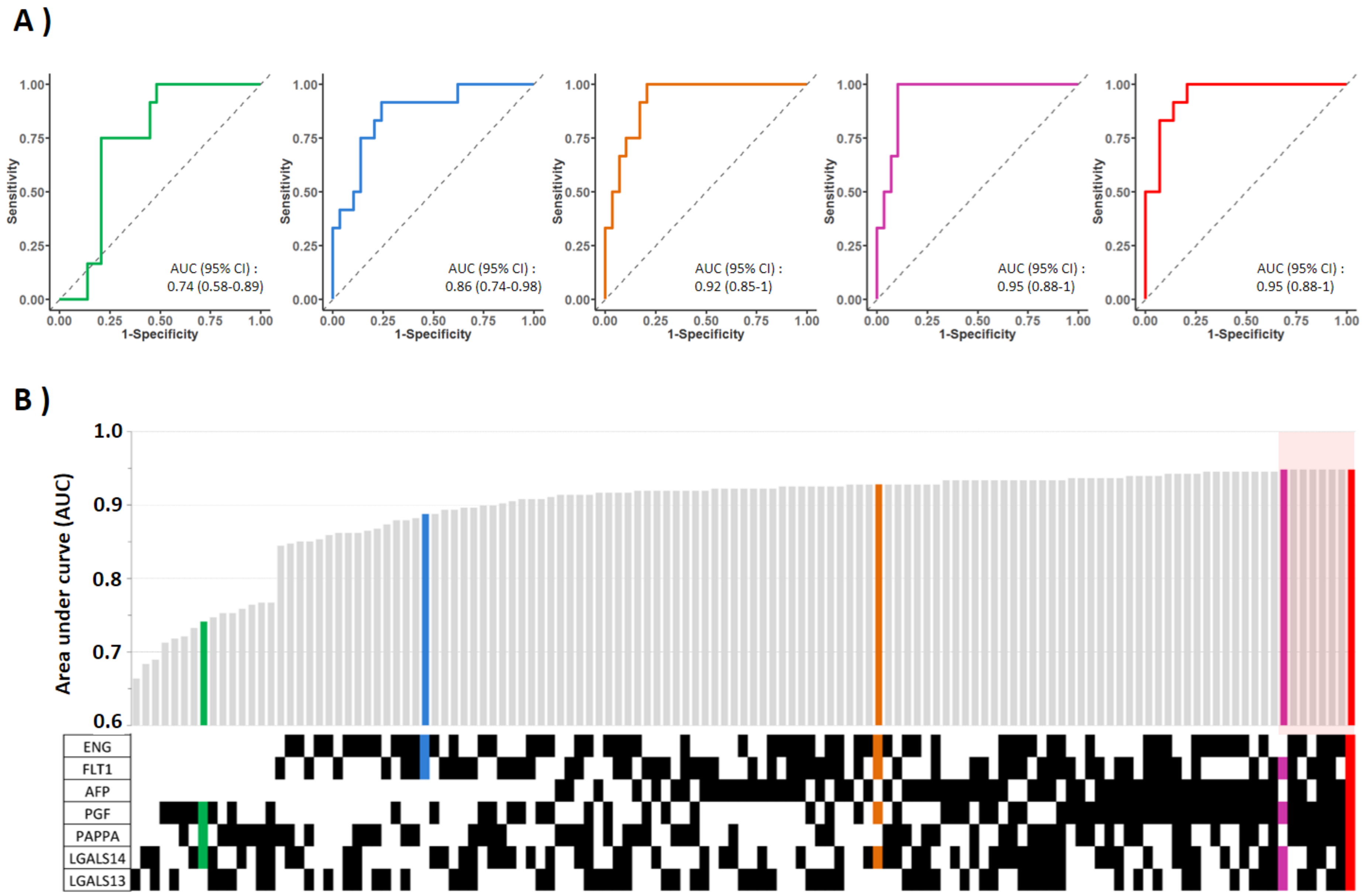
A)
Sensitivity
1-Specificity
1.00
0.75
0.50
0.25
0.00
AUC (95% CI) :
0.74 (0.58-0.89)
AUC (95% CI) :
0.86 (0.74-0.98)
AUC (95% CI) :
0.92 (0.85-1)
AUC (95% CI) :
0.95 (0.88-1)
AUC (95% CI) :
0.95 (0.88-1)
B)
Area under curve (AUC)
1.0
0.9
0.8
0.7
0.6
ENG
FLT1
AFP
PGF
PAPPA
LGALS14
LGALS13
